# Impact of Irradiation on the Pharmacokinetics and Biotransformation of Tamoxifen

**DOI:** 10.3389/fonc.2022.833108

**Published:** 2022-02-17

**Authors:** Yung-Yi Cheng, Teresa Zheng, Michael W. Chang, Jeffrey W. Dalley, Yu-Jen Chen, Tung-Hu Tsai, Chen-Hsi Hsieh

**Affiliations:** ^1^ Division of Pharmacoengineering and Molecular Pharmaceutics, Eshelman School of Pharmacy, the University of North Carolina at Chapel Hill, Chapel Hill, NC, United States; ^2^ Institute of Traditional Medicine, National Yang Ming Chiao Tung University, Taipei, Taiwan; ^3^ Department of Psychology, University of Cambridge, Cambridge, United Kingdom; ^4^ Department of Psychiatry, University of Cambridge, Cambridge, United Kingdom; ^5^ Department of Radiation Oncology, MacKay Memorial Hospital, Taipei, Taiwan; ^6^ Department of Artificial Intelligence and Medical Application, MacKay Junior College of Medicine, Nursing and Management, Taipei, Taiwan; ^7^ Department of Medical Research, China Medical University Hospital, Taichung, Taiwan; ^8^ Department of Medical Research, MacKay Memorial Hospital, New Taipei City, Taiwan; ^9^ Faculty of Medicine, School of Medicine, National Yang Ming Chiao Tung University, Taipei, Taiwan; ^10^ Division of Radiation Oncology, Department of Radiology, Far Eastern Memorial Hospital, New Taipei City, Taiwan

**Keywords:** breast cancer, radiotherapy, tamoxifen, pharmacokinetics, tandem mass spectrometry

## Abstract

**Background:**

The optimal procedure for combining radiotherapy (RT) with tamoxifen treatment is controversial as RT may alter the pharmacokinetics and biotransformation of tamoxifen. The present study investigated this potential interaction by assessing the pharmacokinetics of tamoxifen during concurrent and sequential RT.

**Method:**

Plasma tamoxifen concentration was measured in rats with or without RT 2.0 Gy (RT_2.0Gy_) or 0.5 Gy (RT_0.5Gy_) with ultra-high-performance liquid chromatography-tandem mass spectrometry after tamoxifen administration (10 mg/kg, p.o., *n* = 6). Tamoxifen was either administered 1 h after RT (concurrent condition) or 24 h after RT (sequential condition).

**Results:**

Pharmacokinetic data analysis demonstrated that the area under the curve (AUC) and half-life of tamoxifen were 2,004 ± 241 h ng/ml and 6.23 ± 1.21 h, respectively, after tamoxifen administration (10 mg/kg, p.o.). The respective conversion rate of 4-hydroxytamoxifen, *N*-desmethytamoxifen, and endoxifen for tamoxifen metabolism was 20%, 16%, and 5%. The AUC value of tamoxifen in the RT_0.5Gy_ group was 1.5- to 1.7-fold higher than in the sham and RT_2.0Gy_ groups. The relative bioavailability of tamoxifen at concurrent RT_0.5Gy_ and RT_2.0Gy_ groups ranged from 127% to 202% and from 71% to 152%, respectively. The magnitude of endoxifen, which converted from 4-hydroxytamoxifen and *N*-desmethyltamoxifen, increased 3- to 5-fold in the concurrent RT groups. By contrast, the AUC of tamoxifen decreased by roughly 24% in the sequential RT_2.0Gy_ group. The conversion ratio of endoxifen was four times higher than that in the sequential RT_2.0Gy_ group compared with rats not exposed to RT.

**Conclusion:**

The current study provides advanced pharmacokinetic data to confirm the interaction between RT and hormone therapy. Our findings indicate that RT facilitates the metabolism of tamoxifen to active metabolites and thus imply that combination RT-tamoxifen has potential benefits for the treatment of hormone-dependent breast cancer.

## Introduction

Breast cancer is one of the top 10 diagnosed cancers for women, accounting for 11.7% of total new cases (1). Current treatment strategies for breast cancer include surgery, chemotherapy, endocrine therapy, radiotherapy (RT), immunotherapy, and combinations of these options. The National Surgical Adjuvant Breast and Bowel Project (NSABP) B-06 study demonstrated that the rate of local recurrence after a 20-year follow-up was 39% for patients treated with lumpectomy alone or 14% for patients treated with both lumpectomy and RT (2). The 10-year overall survival of patients was increased for patients who underwent RT, as reported in the MA.20 trial and the European Organization for Research and Treatment of Cancer (EORTC) trial (3, 4).

Four Phase III randomized trials have prospectively evaluated tamoxifen with placebo for breast cancer risk reduction (5–8). The relative risk was reduced by 34%–50% for patients who received 5-year adjuvant tamoxifen therapy (5, 7, 9). Tamoxifen has been prescribed as monotherapy or combined with RT for many years. Ellis and coworkers observed that estrogen-positive breast cells pretreated with tamoxifen had a greater apoptosis index and implies the radiosensitivity of tamoxifen (10). Correspondingly, the NSABP-B14 trial suggested that tamoxifen and RT may have a synergistic interaction since patients receiving both therapies experienced better outcomes in terms of local control (9). Nevertheless, breast cancer patients treated with tamoxifen and RT were 2- to 3-fold more likely to develop radiation-induced pulmonary fibrosis (11, 12). Of note, there were no significant differences in the recurrence, disease-free survival, or overall survival of breast cancer patients treated with tamoxifen concurrent or sequential with RT (13).

RT is associated with reactive oxygen species (ROS) production (14). Moreover, the primary sources of ROS in the liver are the mitochondria and cytochrome P450 enzyme systems and derive from Kupffer and inflammatory cells (15). Additionally, drug pharmacokinetics influenced by RT has previously been revealed (16, 17). Breast cancer patients treated by advanced RT could experience off-target exposure to surrounding organs such as the lung and heart, which might cause unpredictable effects (18, 19). Recently, RT significantly impacted the hepatic microsomal cytochrome P450 3A4 (CYP3A4) activity and P-glycoprotein (P-gp) activity (16, 17). This was likely caused by an unintended interaction between RT and tamoxifen. However, few preclinical studies have specifically investigated the interaction between tamoxifen and RT and how this affects the pharmacokinetics and metabolism of tamoxifen. The present study focused on the RT interaction with tamoxifen and its metabolites using two RT sequences.

## Materials and Methods

### Reagents and Chemicals

Tamoxifen citrate salt (>98%) was obtained from TCI chemicals (TCI, Portland, Oregon, USA). 4-hydroxytamoxifen (>98%), *N-*desmethyltamoxifen hydrochloride (>98%), E/Z endoxifen hydrochloride (>98%) and heparin were obtained from Sigma-Aldrich Chemical Co. (St. Louis, MO, USA). 5-methylflavone (internal standard) was purchased from Tokyo Chemical Industry. LC-MS grade organic solvents, including methanol, acetonitrile, formic acid, and ammonium bicarbonate, were acquired from Merck Co. (Darmstadt, Germany). Purified deionized water was produced using a Milli-Q system (Millipore, Milford, MA, USA).

### Animals

Female Sprague–Dawley rats (200 ± 20 g) were obtained from the Laboratory Animal Centre at National Yang Ming Chiao Tung University (Taipei, Taiwan). Food (Laboratory Rodent Diet 5001, PMI Nutrition International LLC, MO, USA) and water were supplied *ad libitum*. All experimental procedures involving surgery were reviewed and approved by the Institutional Animal Care and Use Committee of National Yang Ming Chiao Tung University (IUCAC no.1051204). For the pharmacokinetic studies, catheterization of the carotid artery was used in unrestrained conscious rats (16). Rats were anesthetized with pentobarbital sodium (50 mg/kg, IP injection, Taoyuan, Taiwan) and implanted with PE-50 polyethylene (I.D. 0.58 mm × O.D. 0.965 mm, MD, USA) tubing in the left carotid artery. The exteriorized catheter was secured in the dorsal neck area and capped with a stopper. Normal heparinized saline (heparin 500 IU/ml in normal saline) was used to maintain the patency of the tubing. Rats were allowed a minimum of 24 h to recover prior to drug administration. Blood samples (150 μl) were collected from the jugular vein at 0.25, 0.5, 1, 2, 4, 6, 8, 10, 12, 16, 18, 20, and 24 h after each drug administration. Plasma was separated by centrifugation at 6000 × g for 10 min at 4°C and stored at −20°C prior to further analysis. After finishing the experiment, an overdose of CO_2_ was used to euthanize animals.

The dose of tamoxifen used daily in humans is typically 20 to 40 mg (20, 21). Based on the human–animal dose conversion formula: human equivalent dose (HED, mg/kg) = animal dose (mg/kg) × animal km (body weight (kg) divided by body surface area (BSA) (m^2^)/human km (22), the range of tamoxifen would then be orally 2.0–4.1 mg/kg in rats. Plasma drug levels are probably below the lowest detectable concentration of quantification, given that the bioavailability of tamoxifen was approximately 20% (23). The tamoxifen dosage orally administered in the present study is consistent with our previous work (24). Experimental animals were randomized into five groups: group 1 (sham group): tamoxifen 10 mg/kg, p.o. plus 0 Gy; two concurrent tamoxifen with single-fraction irradiation; group 2: tamoxifen 10 mg/kg, p.o. plus RT 0.5 Gy (RT_0.5Gy_); group 3: tamoxifen 10 mg/kg, p.o. plus RT 2.0 Gy (RT_2Gy_); sequential tamoxifen with single-fraction irradiation; group 4: tamoxifen 10 mg/kg, p.o. plus RT 0.5 Gy (RT_0.5Gy_), and group 5: tamoxifen 10 mg/kg, p.o. plus RT 2.0 Gy (RT_2Gy_). Rats received tamoxifen 1 hour after RT represented as the concurrent regimen. Receiving tamoxifen 24 h after RT was deemed the sequential regimen. Data were obtained from 6 rats in each group.

### Irradiation Technique

Rats were anesthetized with pentobarbital sodium (50 mg/kg, i.p.) and immobilized on a board to undergo computed tomography to simulate the breast field. The cranial margin was set at the clavicle head and sternum junction with a 2.5 × 4 cm irradiation field. Conventional RT technique was employed to deliver the irradiation dose *via* the anterior portal with a depth of 0.5 cm to the left side chest wall of rats. In the clinical cases treated with different irradiation techniques, more than 50% of the normal liver was exposed to 0.5 Gy (off-target dose) during daily 2-Gy radiation treatments (25). Reviewing several related studies, currently, there is no direct comparison of allometric scaling using chest wall irradiation between humans and rodents. However, the respective lethal dose (LD50) of total-body irradiation for human and rat is 4 and 6.75 Gy (26). The LD50 is defined as the dose that causes a mortality rate of 50% in an experimental group within a specified period of time. Our previous report demonstrated that irradiation of 2 Gy to the rats is safe and workable to stimulate the relevant dose for daily treatment of the human torso. RT 0.5 Gy represented a dose deposited in the off-target area in clinical practice (25). According to the above data, the irradiation doses of 0.5 and 2 Gy for rats simulating the relevant dose range for daily treatment of the human torso were utilized in the current study.

### Sample Preparation

The primary stock solution of tamoxifen, 4-hydroxytamoxifen, *N*-desmethyltamoxifen, and endoxifen was 1 mg/ml in methanol and was stored at −20°C until analysis. The working solutions of calibration curves for quantification analysis were diluted using acetonitrile. For calibration curves, the drug-free plasma (50 μl) and 10-μl working solution were mixed with 140 μl of internal standard solution (acetonitrile containing 50 ng/ml internal standard, ACN-IS solution). The mixed samples were then vortexed for 5 min and centrifuged for 10 min at the speed of 15,000 r.p.m. The collected supernatant was filtered through a 0.22-μm filter before LCMS analysis. Plasma samples were extracted using 150 μl of ACN-IS solution. All prepared samples were kept at 10°C in the autosampler throughout the analysis.

### UPLC-MS/MS for Quantification Analysis

Ultra-high-performance liquid chromatography-tandem triple-quadrupole mass spectrometry (Waters Acquity B.S.M.) equipped with an electrospray ionization device (Waters Xevo TQ MS, Milford, MA, USA) was used in this study to simultaneously determine tamoxifen and its metabolites, including 4-hydroxytamoxifen, *N*-desmethyltamoxifen, and endoxifen. Chromatographic separation was performed by the optimized elution, consisting of 10 mM ammonium bicarbonate and methanol (15/85) with a flow rate of 0.2 ml/min, and using a C18 column (C18, 100 × 2.1 mm, 2.7 μm; Dikama, B.S.M., maintained at 40°C) to accomplish the analysis. The sample injection volume was 5 μl. The MS/MS condition and analyte transition have previously been described (24). The coefficient correlation (*r* ^2^) as the linearity standard was at least 0.995. The relative standard deviation (RSD, %) was precision calculated by RSD (%) = (standard deviation/observed concentration) × 100%. The closeness of a measured value to the mean value for the actual value was accuracy (bias, %), which was calculated as follows: accuracy (%) = [(the nominal concentration − the observed concentration)/the nominal concentration] × 100%. The acceptable value of precision and accuracy was within ±15%, except the lower limit of quantification (LLOQ, defined as a signal-to-noise ratio of less than 10). The precision and accuracy of the LLOQ were always lower than ± 20%.

### Pharmacokinetic Analysis and Statistical Analysis

PK parameters, including the area under the concentration–time curve (AUC), terminal elimination phase half-life (t_1/2_), maximum observed plasma concentration (C_max_), and time of maximum concentration observed (T_max_), were calculated using the PK calculation software WinNonlin Standard Edition, Version 1.1 (Scientific Consulting, Apex, NC, USA). Relative bioavailability (RB %) = (AUC_irradiated_/AUC_control_) × 100. The metabolic conversion of tamoxifen was calculated using the following formula: Metabolite conversion (MC %) = (AUC_metabolite_/AUC_parent_) × 100. The results were presented as means ± standard deviations. Differences in actuarial outcomes between the groups were calculated by one-way analysis of variance (ANOVA) and post-hoc Student–Newman–Keuls tests using the SigmaPlot program (Systat Software Inc., version 6.0). A *p-*value of <0.05 was considered statistically significant.

## Results

### Validation of the UPLC-MS/MS Method

To optimize peak shape and resolution, the composition of the mobile phase and analytical method was carefully adjusted. The robust UPLC-MS/MS-based analytic method to determine the blood concentration of tamoxifen and its major metabolites was established and validated. The retention time of tamoxifen, 4-hydroxytamoxifen, *N*-desmethyltamoxifen, and endoxifen individually were 4.4, 2.3, 3.2, and 1.9 min, respectively. The linear concentration ranges with acceptable coefficient correlation (*r* ^2^ > 0.995) of tamoxifen, 4-hydroxytamoxifen, *N*-desmethyltamoxifen, and endoxifen were 1–1,000 ng/ml. The accuracy and precision data of the relevant concentration ranges enabled effective quantification. The accuracy and precision of intra-day ranged from 0.15 to 15.20 and from −13.52 to 12.78, respectively. The accuracy and precision of inter-day were within 0.16–13.12 and −13.48–7.04, respectively. The accuracy and precision of the relevant concentration ranges were thus acceptable for quantification.

### Pharmacokinetics and Biotransformation of Tamoxifen in Plasma

**Figure 1:**
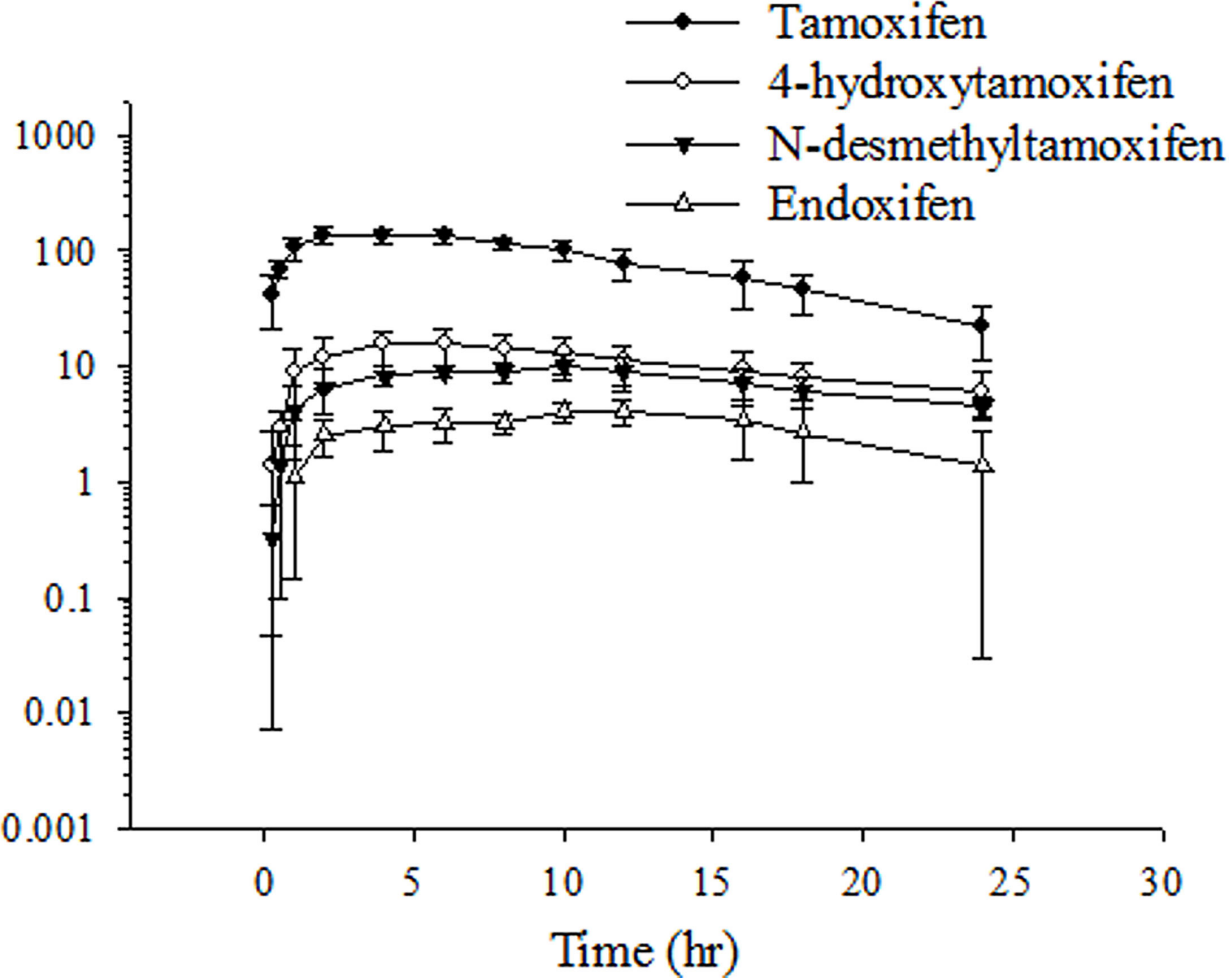
Time–concentration curve of tamoxifen, 4-hydroxytamoxifen, *N*-desmethyltamoxifen, and endoxifen in rat plasma after oral tamoxifen administration (10 mg/kg). Data are expressed as the mean ± standard deviation (*n* = 6 per group).

The profile of the plasma concentration–time curves of tamoxifen and its metabolites after female rats single orally administered tamoxifen is illustrated in **Figure 1**. Tamoxifen reached the C_max_ (143.9 ± 25.2 ng/ml) after approximately 5.5–8.6 h (T_max_) after orally receiving tamoxifen. A non-compartmental model was used to analyze the pharmacokinetic data. The pharmacokinetic parameters of tamoxifen and its metabolites are summarized in **Table 1**. The AUC of tamoxifen, 4-hydroxytamoxifen, *N*-desmethyltamoxifen, and endoxifen was 2,004 ± 241, 385.7 ± 149.8, 283.7 ± 61.8, and 92.7 ± 44.2 h ng/ml, respectively. The half-lives of tamoxifen, 4-hydroxytamoxifen, *N*-desmethyltamoxifen, and endoxifen was 6.23 ± 1.21, 13.6 ± 6.4, 16.4 ± 10.4, and 7.48 ± 2.57 h, respectively. Additionally, the biotransformation of tamoxifen was evaluated by metabolic conversion, calculated by the metabolite-to-tamoxifen AUC ratio for 4-hydroxytamoxifen, *N*-desmethyltamoxifen, and endoxifen. Based on the formula of metabolic conversion, the metabolic conversion of 4-hydroxytamoxifen, *N*-desmethyltamoxifen, and endoxifen was 19.6 ± 8.1%, 16.1 ± 2.4%, and 4.73 ± 2.27%, respectively. The metabolic conversion of 4-hydroxytamoxifen ranged from approximately 20% and was compatible with previous studies (24). However, the related studies rarely investigated the metabolic conversion of *N*-desmethyltamoxifen and endoxifen. Our present study showed the magnitude of 5% conversion of endoxifen for tamoxifen metabolism, and 4-hydroxytamoxifen and *N*-desmethyltamoxifen had a similar ratio of conversion.

**Table 1:** The parameters of pharmacokinetic in tamoxifen, 4-hydroxytamoxifen, *N*-desmethyltamoxifen, and endoxifen for rats treated with tamoxifen (10 mg/kg, p.o.) concurrent or sequential with irradiation 0.5 Gy (RT_0.5Gy_) and 2 Gy (RT_2.0Gy_).

	Tamoxifen (10 mg/kg, p.o.)				
Pharmacokinetic parameters	Sham	Concurrent		Sequential	
	0 Gy	0.5 Gy	2.0 Gy	0.5 Gy	2.0 Gy
Tamoxifen					
AUC (h ng/ml)	2,004 ± 241	3,468 ± 722	2,089 ± 582	2,021 ± 299	1,530 ± 167*^# 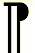 ^
t_1/2_ (h)	6.23 ± 1.21	5.54 ± 1.10	7.14 ± 1.85	5.85 ± 1.30	5.28 ± 1.66
T_max_ (h)	4.67 ± 1.63	5.00 ± 1.09	8.00 ± 2.83	4.17 ± 2.23	5.67 ± 2.66
C_max_ (ng/ml)	143.9 ± 25.2	270.4 ± 66.3	135.8 ± 38.9	156.6 ± 43.7	108.0 ± 13.9
Relative bioavailability (%)	100	173.0 ± 36.1	104.3 ± 29.1	107.3 ± 21.7	76.4 ± 8.3*^# 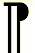 ^
4-Hydroxytamoxifen					
AUC (h ng/ml)	385.3 ± 149.8	705.1 ± 103.0	526.9 ± 138.7	524.0 ± 130.0	331.2 ± 37.8
t_1/2_ (h)	13.6 ± 6.4	6.59 ± 1.88	7.59 ± 2.07	7.05 ± 3.09	5.79 ± 5.10
T_max_ (h)	5.0 ± 1.1	5.33 ± 1.03	9.33 ± 2.07	5.67 ± 0.82	8.33 ± 2.33
C_max_ (ng/ml)	16.9 ± 5.1	44.0 ± 8.3	29.3 ± 8.1	34.9 ± 9.8	21.3 ± 4.4
Metabolic conversion (%)	19.6 ± 8.1	21.2 ± 5.99	25.2 ± 6.6	25.7 ± 5.0	21.8 ± 3.3
*N*-Desmethyltamoxifen					
AUC (h ng/ml)	283.7 ± 61.8	743.7 ± 81.9	354.9 ± 96.4	460.7 ± 139.5	243.2 ± 40.7
t_1/2_ (h)	16.4 ± 10.4	12.3 ± 9.2	11.4 ± 2.0	8.09 ± 2.11	5.67 ± 3.40
T_max_ (h)	9.33 ± 2.07	8.00 ± 4.56	10.3 ± 2.3	8.67 ± 2.73	9.67 ± 2.66
C_max_ (ng/ml)	11.9 ± 2.7	33.7 ± 12.4	17.1 ± 6.3	23.2 ± 9.5	15.1 ± 2.1
Metabolic conversion (%)	16.1 ± 2.4	22.2 ± 4.9	17.2 ± 3.3	22.4 ± 3.9	15.9 ± 2.2
Endoxifen					
AUC (h ng/ml)	92.7 ± 44.2	546.9 ± 203.8*	454.9 ± 103.1*	449.1 ± 127.9*	258.6 ± 52.8*^# 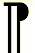 ^
t_1/2_ (h)	7.48 ± 2.57	11.8 ± 3.5	10.2 ± 3.9	9.74 ± 3.98	13.4 ± 22.7
T_max_ (h)	8.00 ± 4.00	11.0 ± 4.3	9.67 ± 2.33	12.5 ± 4.6	12.3 ± 3.2
C_max_ (ng/ml)	4.77 ± 1.36	21.7 ± 6.2	20.2 ± 4.0	19.9 ± 5.1	13.6 ± 4.4
Metabolic conversion (%)	4.73 ± 2.27	16.8 ± 7.2*	23.0 ± 8.3*	22.0 ± 4.4*	17.1 ± 4.1*

AUC_0-∞_: area under the plasma concentration–time curve from 0 h to infinity; C_max_: peak plasma concentration; t_max_: time to reach C_max_; t_1/2_: terminal half-life. *p < 0.05, a significant difference compared with the sham group within the group by one-way ANOVA and Student–Newman–Keuls post-hoc tests. ^#^p < 0.05, a significant difference compared with the concurrent RT_2.0Gy_ group. ^
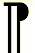
^p < 0.05, a significant difference compared with the sequential RT_0.5Gy_ group. Data are expressed as the mean ± standard deviation (n = 6 per group).

### Pharmacokinetics and Biotransformation of Tamoxifen During the Concurrent Regimen

**Figure 2:**
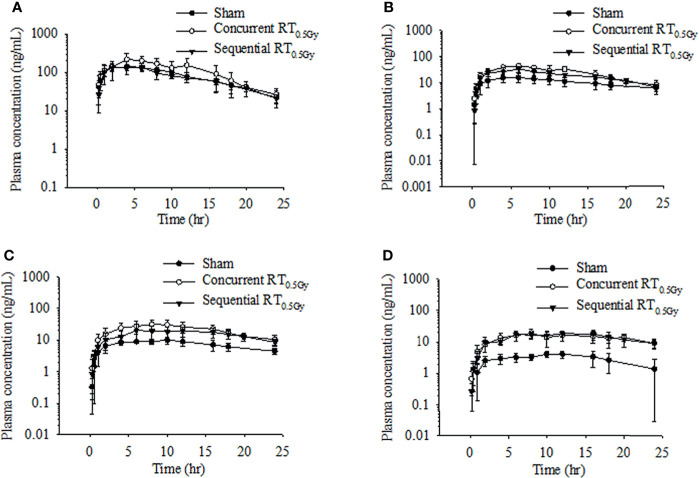
Time–concentration curve of tamoxifen **(A)**, 4-hydroxytamoxifen **(B)**, N-desmethyltamoxifen **(C)**, and endoxifen **(D)** in rat plasma after oral administration at the dose of RT_0Gy_ or RT_0.5 Gy_. (●) Sham group: tamoxifen administration (10 mg/kg, p.o.) plus RT_0Gy_; (○) Concurrent RT_0.5Gy_ group: tamoxifen administration (10 mg/kg, p.o.) plus RT_0.5Gy_; (▼) Sequential RT_0.5Gy_ group: tamoxifen administration (10 mg/kg, p.o.) plus RT_0.5Gy_. Data are expressed as the mean ± standard deviation (*n* = 6 per group).

**Figure 3:**
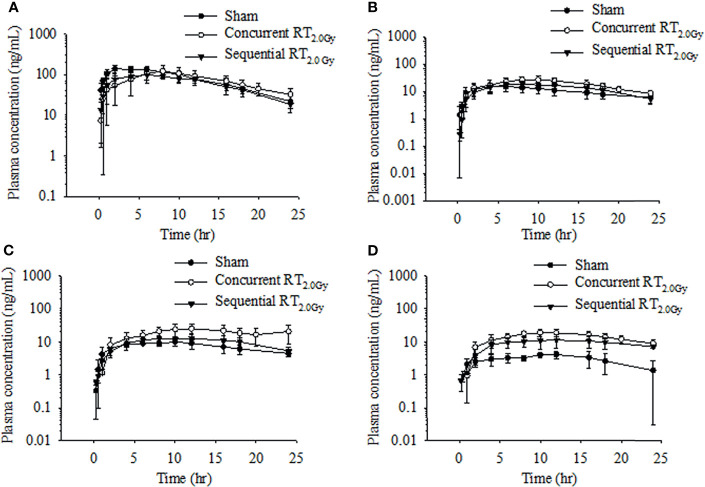
Time–concentration curve of tamoxifen **(A)**, 4-hydroxytamoxifen **(B)**, N-desmethyltamoxifen **(C)**, and endoxifen **(D)** in rat plasma after oral administration at the dose of RT_0Gy_ or RT_2.0 Gy_. (●) Sham group: tamoxifen administration (10 mg/kg, p.o.) plus RT_0Gy_; (○) Concurrent RT_2.0Gy_ group: tamoxifen administration (10 mg/kg, p.o.) plus RT_2.0Gy_; (▼) Sequential RT_2.0Gy_ group: tamoxifen administration (10 mg/kg, p.o.) plus RT_2.0Gy_. Data are expressed as the mean ± standard deviation (*n* = 6 per group).

Rats received tamoxifen 1 h after radiation (concurrent regimen) to determine effects on pharmacokinetic variables in the sham, RT_0.5Gy_, and RT_2.0Gy_ groups. Plasma concentration curves are illustrated in **Figures 2** and **3**. The relevant non-compartmental parameters are listed in **Table 1**. At the RT_0.5Gy_ group, the C_max_ of tamoxifen and *N*-desmethyltamoxifen was 2-fold higher than the respective C_max_ at the sham and RT_2.0Gy_ groups. The AUC value in the RT_0.5Gy_ group was 1.5- to 1.7-fold higher than in the sham and RT_2.0Gy_ groups. As shown in **Table 1**, the relative bioavailability of tamoxifen was higher in RT_0.5Gy_ (ranging from 127% to 202%) than in RT_2.0Gy_ (ranging from 71% to 152%). Regarding the relevant PK parameters, half-lives and T_max_ were not significantly different from the sham group. However, compared to the concurrent irradiated groups with the sham group, the metabolic conversion of *N*-desmethyltamoxifen and endoxifen was significantly increased. The 4-hydroxytamoxifen conversion ratio was not significantly affected by the concurrent regime. Interestingly, however, the magnitude of endoxifen converted from 4-hydroxytamoxifen and *N*-desmethyltamoxifen increased 3- to 5-fold.

### Pharmacokinetics and Biotransformation of Tamoxifen During the Sequential Regimen

In this condition, tamoxifen was administered 24 h after irradiation with either 0.5 Gy or 2.0 Gy. The mean plasma concentration–time profile following single oral administration of tamoxifen with RT_0.5Gy_ and RT_2.0Gy_ is shown in **Figures 2** and **3**. There were no significant differences in AUCs between the sham and sequential RT_0.5Gy_ groups. Additionally, the metabolic conversion for 4-hydroxytamoxifen and *N*-desmethyltamoxifen was similar in the sequential RT_0.5Gy_ and sham groups except for endoxifen. Nevertheless, the AUC of tamoxifen in the sequential RT_2.0Gy_ group decreased by 24%; the relative biotransformation (%) was decreased by approximately 76%, compared to the sham group. Regarding metabolic conversion in the RT_2.0Gy_ treatment, the conversion level of 4-hydroxytamoxifen and *N*-desmethyltamoxifen was not significantly affected. However, for the RT_2.0Gy_ group, endoxifen conversion was four times higher than that of the non-irradiated group. There were no other significant effects of RT on the various PK parameters.

The change of tamoxifen metabolites between the concurrent and sequential regimens was analyzed. The respective AUC of 4-hydroxytamoxifen increased by 83% and 37% in the concurrent RT_0.5Gy_ and RT_2.0Gy_ groups, respectively. By 36% of the increase in the sequential RT_0.5Gy_ group. In the concurrent RT_0.5Gy_ and RT_2.0Gy_ groups, the AUC of endoxifen increased by 488% and 389%, respectively; the respective metabolic conversion ratio of endoxifen for tamoxifen concurrent with RT_0.5Gy_ and RT_2.0Gy_ was 3.4- and 4.6-fold higher than the sham group. Furthermore, the AUC of endoxifen in the sequential regimen increased in the RT_0.5Gy_ group, by 383% and 178%, respectively. The metabolic conversion rate of endoxifen for tamoxifen sequential with RT_0.5Gy_ and RT_2.0Gy_ was 4.4- and 3.4-fold that of the sham group, respectively.

## Discussion

The biochemical pathways responsible for tamoxifen metabolism are complex, involving recently discovered estragon-like tamoxifen metabolites and cytochrome P450-dominated processes, particularly CYP3A4 CYP2D6 isomers (27). In short, tamoxifen is metabolized from 4-hydroxylated to 4-hydroxytamoxifen by CYP2D6 isomers and from *N*-dealkylated to *N*-desmethyltamoxifen by CYP3A4 isomers. *N*-desmethyltamoxifen and 4-hydroxytamoxifen individually undergo these reactions to form the secondary metabolites, *N*, *N*-didesmethyltamoxifen, and endoxifen (27). Among them, 4-hydroxytamoxifen and endoxifen modulate the therapeutic efficacy of tamoxifen due to their competitive binding to estragon receptors with a binding affinity 30–100 times higher than tamoxifen itself, resulting in the inhibition of breast tumor growth (27, 28).

According to the Surveillance, Epidemiology, and End Results (SEER) data from 2005 to 2010, the incidence for male breast cancer was 2,054 male/289,673 all breast cancer cases (29). Male breast cancer is almost hormone receptor positive, and the use of endocrine therapy such as tamoxifen is routine for managing male breast cancer (30). In comparing our previous study (24) and the current study, the AUC of tamoxifen in female rats was about 1.2-fold higher than that in male rats. Moreover, the metabolic conversion ratio of 4-hydroxytamoxifen to *N*-desmethyltamoxifen in female rats was roughly onefold and the ratio of 4-hydroxytamoxifen to endoxifen conversion was 4- to 5-fold. However, our previous study demonstrated that the percentage of 4-hydroxytamoxifen conversion in male rats is only 26% (24). The endoxifen conversion ratio in female rats is significantly greater than that in male rats, implying that the endoxifen conversion ratio is higher in female rats than in male rats.

It has been reported that RT affects the cytochrome P450 enzyme, resulting in the change of drug pharmacokinetics (16, 31). Consequently, in the present study, we investigated the RT-tamoxifen combination on pharmacokinetic behavior and biotransformation. As **Figure 3** illustrated, the T_max_ of tamoxifen was 3 to 4 h more prolonged in the concurrent RT_2.0Gy_ group compared with the sham group. Conversely, in the sequential RT_2.0Gy_ group, no significant effects were observed. As previously discussed, endoxifen and 4-hydroxytamoxifen have an approximately 100-fold greater affinity for the estrogen receptor (ER) and the ability to inhibit cell proliferation. Additionally, 4-hydroxytamoxifen has been shown to cause specific ER-mediated and non-specific ER-independent cytotoxic effects in a dose-dependent manner (32). Moreover, endoxifen exhibits superior antiestrogenic activity than the parent drug (33), and the concentration of endoxifen is probably responsible for the clinical outcomes of breast cancer patients receiving adjuvant tamoxifen treatment (34).

Recent NSABP-B14 and NSABP B-24 trials reported that concurrent tamoxifen with RT has a higher probability of local control for patients with breast cancer (9, 35). However, sequential tamoxifen treatment following RT is more effective than concurrent treatment (36). Moreover, the addition of RT to adjuvant tamoxifen reduces the number of in-breast recurrences for older women from 80 to 20 (95% CI 10–40) per 1,000 patients at 10 years (37). Interestingly, in a recent prospective randomized study comparing breast pain after breast-conserving surgery plus tamoxifen with or without RT (38), the incidence and severity of breast symptoms were similar at baseline in patients subsequently randomized to the RT and no-RT arms. Nevertheless, Azria D and colleagues reported that concomitant use of tamoxifen with RT increases the incidence of grade 2 subcutaneous fibrosis in hypersensitive patients (39). Also, the risk of pulmonary fibrosis is increased by 20% for breast cancer patients treated with concurrent regimens (11, 12).

According to our findings, the AUC of endoxifen was significantly enhanced in the RT_2.0Gy_ and RT_0.5Gy_ groups. Endoxifen is converted from *N*-desmethyltamoxifen by the critical phase I enzyme, CYP2D6, responsible for the endoxifen level (40). Moreover, previous evidence has shown that poor and ultrarapid CYP2D6 metabolizers of tamoxifen could predict worse clinical outcomes among patients with breast cancer treated with tamoxifen (41). The association between CYP2D6*4 and radiation toxicity has been reported by Damaraju and coworkers (42). However, CYP2D6*4 is one of the typical CYP2D6 inactive alleles with nonfunctional CYP2D6 activity (27). Our pharmacokinetic profiles and results implied that the 4-hydroxytamoxifen, *N*-desmethyltamoxifen, and endoxifen levels are enhanced in both the irradiated 2.0-Gy groups. In the RT_2.0Gy_ groups, the increasing magnitude of the sequential regimen is slightly lower than the concurrent regimen. Significantly, the endoxifen level and conversion ratio after RT were increased compared with the sham group. Based on these findings, local chest wall irradiation with both 0.5 and 2 Gy probably influences the cytochrome P450 family activity, resulting in altered drug concentration in plasma and drug biotransformation. Additionally, the similar trend of endoxifen converted by tamoxifen in the concurrent and sequential regimens with RT 0.5 and 2 Gy may partly explain why both regimens provide effective clinical outcomes (9, 35, 36).

Interestingly, the AUC levels of tamoxifen and metabolites in the sequential regimen were opposite for the RT_2.0Gy_ and RT_0.5Gy_ groups. Compared with the sequential group, the AUC for tamoxifen in the sham group increased by 1% in the RT_0.5Gy_ group but decreased by 24% in the RT_2.0Gy_ (*p* < 0.05). These data imply that the tamoxifen may be similarly affected by off-target irradiation as well as non-irradiated group during the sequential regimen and may be exposed under a tamoxifen level of similar magnitude to that of the non-irradiated group. On the contrary, as **Figure 2** shows, in the concurrent RT_0.5Gy_ group, the AUC levels of tamoxifen are appreciably more significant than that in the concurrent RT_2.0Gy_ and sham groups. However, there were no differences in AUC levels between the concurrent RT_2.0Gy_ and sham groups. Higher concentrations of tamoxifen or toxic metabolites may lead to adverse effects or better control (28). Therefore, the relatively higher tamoxifen level in the concurrent RT_0.5Gy_ group may be considered a consequence of irradiation to the off-target area.

Recently, it was found that protein expression and activity could be changed by doses of RT dose as low as 1.0 Gy (43). RT may impair the vascular and lymphatic systems, thus causing endothelial cell loss (44), which has been associated with enhanced vascular permeability that may enhance the drug’s easier permeation into the lesion from blood circulation and can effectively reach the target despite lower tamoxifen levels. In the current study, tamoxifen translated to endoxifen was increased even after being irradiated 0.5 Gy. The findings imply that the irradiated surrounding normal tissues tolerated the high tamoxifen level during the concurrent regimen. However, tamoxifen has a narrow therapeutic window. Either the extreme change of drug level or the high-dose exposure under the narrow-therapeutic index drugs probably enhances the cytotoxicity to the surrounding normal tissues. These lines of PK’s data support the conversional effect of tamoxifen and could be modulated by RT in both concurrent and sequential regimens. More importantly, the current data also point out the potential toxicity caused by off-target doses for patients with breast cancer treated with advanced RT techniques and tamoxifen.

Most breast cancer tumors are generally considered immunologically “cold”, with low immune cell infiltration, and are highly difficult to target with immunotherapy (45). Recently, Wolfson and colleagues (46) demonstrated that tamoxifen and 4-hydroxytamoxifen sensitize breast cancer cells to natural killer (NK) cell-mediated killing as immunomodulatory agents regardless of estrogen receptor expression. Additionally, G protein-coupled receptor 30 (GPR30, also known as G protein-coupled estrogen receptor, GPER) binds estrogen (47) and as ligands for tamoxifen (48) and 4-hydroxytamoxifen (49). Moreover, GPR30 has also been shown to be associated with extracellular signal-regulated kinase-mediated phosphatidylinositol 3-kinase (PI3K)/serine/threonine kinase Akt signaling pathways (50). Moreover, RT induced ROS generation and nuclear factor-κB activation, and facilitated polymorphonuclear cell leukocyte accumulation (51) that influences the immune response (52). Intriguingly, RT activates mitogen-activated protein kinase and PI3K pathway (53). These lines of evidence support a rationale for further investigation of combination with ER targeting drugs, immuno-oncology agents, and RT.

There were some limitations to our study. First, the current study was designed to examine the interaction between RT and the PK of tamoxifen, but it does not include the pharmacodynamics of tamoxifen during RT. However, the published clinical data support the synergistic effect of treatment and toxicity. Additionally, the current analysis sheds light on the discrepancies of PK in the concurrent and sequential regimens of RT with tamoxifen, which will be helpful in the clinical setting. Second, the current study used a single fraction instead of multiple daily fractions to investigate the interaction between RT and tamoxifen. Third, the current study used normal and healthy rats instead of rats with breast cancer disease model. However, the present findings addressed the basic pharmacokinetic interaction between radiation and tamoxifen that established the foundation of the relevant disease models. Although these limitations are present, our previous study suggested that the AUC of drugs could be influenced by multiple fractions and a single fraction (31). Hence, the interaction of tamoxifen between multiple fractions model is warranted in the future. The possible mechanism was not examined in the current study because the presence or absence of the RT-PK phenomenon in the context of tamoxifen plus RT could not be ensured before the study. After the study, the RT-PK phenomenon of tamoxifen is confirmed, and the possible mechanism of the disease model should be examined in the future.

To our best knowledge, the current study is the first to confirm the RT–drug interaction with RT-PK phenomenon between RT, tamoxifen, and metabolites. RT could modulate the systemic PK of tamoxifen and metabolites with off-target and treatment doses with different combinations of regimens. These findings provide the rationale for further studies to investigate the interactive effects of RT on the pharmacokinetics and biotransformation of tamoxifen.

## Data Availability Statement

The original contributions presented in the study are included in the article/supplementary material. Further inquiries can be directed to the corresponding authors.

## Ethics Statement

The animal study was reviewed and approved by IUCAC no. 1051204. Written informed consent was obtained from the owners for the participation of their animals in this study.

## Author Contributions

Designed and executed the research, Y-YC. Prepared the original draft, Y-YC, TZ, MC, and C-HH. Analyzed the research data, TZ, MC, and Y-YC. Editing the manuscript, Y-YC, C-HH, JD, and T-HT. Supervision, project administration, and funding acquisition, C-HH, Y-JC, and T-HT. All authors contributed to the article and approved the submitted version.

## Funding

This work was supported in part by grants from Far Eastern Memorial Hospital (FEMH 110-2314-B-418-006), the Ministry of Science and Technology, Taiwan (MOST 110-2314-B-418-006; MOST 110-2981-I-239-001; MOST 110-2113-M-A49A-503; MOST 109-2314-B-195-003-MY3), the MacKay Memorial Hospital (MMH-E-110-13; MMH-E-111-11), and the NYMU-FEMH Joint Research Program (110DN38).

## Conflict of Interest

The authors declare that the research was conducted in the absence of any commercial or financial relationships that could be construed as a potential conflict of interest.

The reviewer YCH declared a shared affiliation to the with one of the authors, YJC, to the handling editor at time of review.

## Publisher’s Note

All claims expressed in this article are solely those of the authors and do not necessarily represent those of their affiliated organizations, or those of the publisher, the editors and the reviewers. Any product that may be evaluated in this article, or claim that may be made by its manufacturer, is not guaranteed or endorsed by the publisher.
